# Practice patterns in myeloma bone disease: a multi-center survey

**DOI:** 10.3389/fonc.2026.1948667

**Published:** 2026-10-14

**Authors:** Timothy Schmidt, Ryan Gennette, Craig Cole, Aimee Merino, Danai Dima, Malini Surapaneni, Hira Shaikh, Elvira Umyarova, Christopher D’Angelo, Matias Sanchez, Kevin Rakszawski, Natalie Callander, Matthew Pianko, Attaya Suvannasankha

**Affiliations:** 1 University of Wisconsin-Carbone Cancer Center, University of Wisconsin-Madison, Madison, WI, United States; 2 Department of Medicine, Michigan State University, East Lansing, MI, United States; 3 Department of Medicine, University of Minnesota, Minneapolis, MI, United States; 4 Division of Hematology and Oncology, United States, University of Washington, Seattle, WA, United States; 5 Department of Hematology-Oncology, Indiana University, Bloomington, IN, United States; 6 Department of Internal Medicine, University of Iowa, Iowa City, IA, United States; 7 Department of Internal Medicine, Ohio State University, Columbus, OH, United States; 8 Division of Hematology, University of Nebraska Medical Center, Omaha, NE, United States; 9 Division of Hematology/Oncology, University of Illinois-Chicago, Chicago, IL, United States; 10 Department of Internal Medicine, Penn State Cancer Institute, Pennsylvania State University, Hershey, PA, United States; 11 Department of Internal Medicine, University of Michigan, Ann Arbor, MI, United States

**Keywords:** Bisphosphonates (BP), multiple myeloma, myeloma bone disease, Skeletal related events (SREs), supportive care, survey

## Abstract

**Background:**

Bone disease is a common manifestation of multiple myeloma. Although anti-resorptive therapy is recommended by guidelines, implementation can be variable. We sought to evaluate practice patterns for myeloma bone disease management and to identify opportunities for harmonization.

**Methods:**

An online survey was distributed to clinicians who see patients with multiple myeloma at medical centers affiliated with the Big Ten Cancer Research Consortium. Anonymous responses were collected regarding self-reported demographics, as well as practice patterns regarding preferred antiresorptive agent, dosing, duration of therapy, therapy modifications, vitamin and mineral supplementation, dental restrictions, and management at relapse.

**Results:**

A total of 100 responses were collected from 11 different academic institutions and their community-affiliated sites. Respondents were predominantly physicians (74%) working in an academic setting (77%), with variable time practicing independently and number of myeloma patients seen per year. Most respondents (89%) reported using zoledronic acid as the preferred agent, with every 4 weeks as the most common frequency (for zoledronic acid 70%, denosumab 64%), with most administering therapy for 2 years (72%). Most clinical scenarios did not affect anti-resorptive choice at diagnosis, except for severe renal insufficiency. Dental recommendations were heterogeneous. There was low utilization of biomarkers to guide management decisions.

**Conclusion:**

Our survey suggests that at the time of diagnosis, most clinicians administer appropriate supportive care for myeloma bone disease. Further study is needed to help unify practice patterns in the relapsed setting, when dental care is suboptimal, and when it is appropriate to tailor such therapy.

## Introduction

Multiple myeloma (MM) is the second most common hematological malignancy, and a majority of patients are afflicted with bone disease, including lytic lesions, pathologic fractures, and decreased bone density (1). Although great strides have been made to improve long-term outcomes among patients with MM, bone disease has a great impact on patients’ mobility, performance status, and overall quality of life (2). While anti-myeloma therapy helps to limit the progression of myeloma bone disease (MBD), the initial damage can be irreversible in many cases. Patients often remain at risk for skeletal-related events (SRE) and osteoporosis complications for the remainder of their lives even with well-controlled disease. As such, management of patients with MM should include supportive measures to reduce the morbidity of MBD.

A key mechanism that drives the development of MBD is through dysregulation of complex bone remodeling processes, leading to increased bone resorption by overactivated osteoclasts and inhibition of osteoblasts within the bone marrow microenvironment (3). Additionally, osteoclasts may be manipulated by myeloma cells to facilitate disease progression and confer resistance to therapies (4–6). Bisphosphonate therapy has, therefore, been a mainstay of MM supportive care for decades, ever since pamidronate was shown to reduce the rate of SREs compared to placebo (7). Zoledronic acid subsequently became a preferred bisphosphonate after demonstrating reduced skeletal morbidity and improved survival compared with clodronate in the Myeloma IX trial, although this study preceded contemporary imaging and anti-myeloma therapies (8). Due to its more convenient administration, zoledronic acid is often utilized over pamidronate (9). Denosumab, a RANKL inhibitor, was subsequently evaluated and found to be non-inferior to zoledronic acid, with a different toxicity profile (10).

While bisphosphonates and denosumab are highly effective at reducing the risk of SREs in MM, their use is complicated by a variety of toxicities and logistical considerations. All can cause hypocalcemia, especially in the setting of vitamin D deficiency (11), which is common among patients with MM (12). Bisphosphonates are renally cleared and zoledronic acid, in particular, can cause or exacerbate renal dysfunction (13). Dose and schedule modifications are therefore recommended for patients with renal dysfunction who are receiving bisphosphonates to mitigate this risk. Osteonecrosis of the jaw (ONJ) is an uncommon but potentially serious complication of bisphosphonates and RANKL inhibitors. In clinical trials, ONJ has been reported in 3-4% of patients receiving denosumab and zoledronic acid, although some reports state the risk may be up to 10% (10). Many practitioners request dental evaluation prior to using anti-resorptive therapy, but due to cost or unavailability of dental providers, this is often not feasible for patients.

The International Myeloma Working Group (IMWG), the American Society of Clinical Oncology (ASCO), National Comprehensive Cancer Network (NCCN), and European Hematology Association/European Myeloma Network (EHA-EMN) have published evidence-based guidelines regarding the management of MBD (14–16). These evidence-based guidelines provide a very useful framework to navigate this complicated topic. However, adherence to these guidelines is often difficult for oncologists due to the heterogeneous nature of MM, provider opinions, patient-specific factors, and logistical concerns. Therefore, an assessment of practice patterns for the management of MBD may help to identify common themes that could be important when considering strategies to improve and preserve bone health among patients with MM.

Furthermore, although modern anti-myeloma therapies are highly effective, only proteasome inhibitors have demonstrated direct bone anabolic effects (17). Therefore, the risk of skeletal-related events persists and accumulates over time, especially in our growing population of older and often frail long-term survivors. In an era where myeloma is among the most expensive cancers to treat (18), survivorship issues like bone health can be overlooked. This study underscores the critical need to maximize the utility of our supportive care to mitigate the burden of myeloma bone disease.

## Methods

We designed an online survey intended to assess practice patterns among clinicians for management of MBD and distributed the survey to sites affiliated with the Big Ten Cancer Research Consortium (BTCRC) Myeloma Working Group. Most BTCRC sites have a primary academic research hospital, as well as a variety of satellite clinics in the region that operate as community oncology partners. This framework allows for the collection of data from practitioners in both academic and community type settings.

Each participating BTCRC center designated a “site champion” who was responsible for identifying potential survey respondents within their affiliated hospital network. Respondents were eligible to complete the survey if they considered themselves to be involved in management decisions for patients with MM at either the main hospital location or affiliated community cancer centers. Pre-specified clinical roles for survey participants included physicians, advanced practice providers, pharmacists, and others. The survey and recruitment materials were approved as minimal risk by the University of Wisconsin Institutional Review Board. Questions were uploaded to the University of Wisconsin Qualtrics survey tool and disseminated via email to the site champions, who then distributed the survey with an anonymous link to eligible respondents within each BTCRC site network.

Responses were recorded between January 10, 2025 – March 29, 2025. This constituted a time period after the survey was approved for distribution, and the survey was closed when each participating site was no longer routinely receiving responses from potential candidates. There were no incentives provided to respondents, although middle authorship order for the site champions was determined by the number of responses received by respondents affiliated with their institution. Survey questions included a brief selection of self-reported demographic information, with clinical questions asking about clinician preference about choice of anti-resorptive agent, frequency and duration of administration, factors that impact whether to deviate from this preference, and supplemental care, in both newly diagnosed and relapsed/refractory settings. A complete list of survey questions and possible answers is provided in the **Supplementary Appendix**. Figures were generated using IBM SPSS Statistics version 29.0.1.0.

## Results

Of 114 survey webpage visits, 100 individuals (87.7%) recorded survey responses. Self-reported demographic characteristics of the respondents are summarized in **Table 1**. 77% of respondents were physicians and 74% practiced at an academic center, with a wide variety of clinical experience, sub-specialization, and geographic location.

**Table 1:** Respondents’ characteristics.

Characteristic	N	%
Professional role		
Physician	77	77.0%
APP	16	16.0%
Pharmacist	7	7.0%
Practice setting		
Academic	74	74.0%
Academic-affiliated community practice	17	17.0%
Community/private practice	9	9.0%
Time in independent practice		
0-5 years	43	43.0%
5-10 years	25	25.0%
10-20 years	17	17.0%
20+ years	15	15.0%
Myeloma patients seen per year		
0-5	6	6.0%
5-20	30	30.0%
20-50	21	21.0%
50-100	12	12.0%
100+	31	31.0%
BTCRC University Affiliation		
Michigan State University	16	16.0%
University of Wisconsin	14	14.0%
University of Washington	13	13.0%
University of Minnesota	13	13.0%
Indiana University	11	11.0%
University of Michigan	10	10.0%
University of Iowa	9	9.0%
Ohio State University	6	6.0%
University of Nebraska	4	4.0%
University of Illinois	2	2.0%
Penn State University	2	2.0%

When presented with a case of an index patient with newly diagnosed myeloma, manifesting with scattered lytic bone lesions and anemia, without renal dysfunction or hypercalcemia, 89% of respondents preferred zoledronic acid over denosumab (9%) (**Figure 1a**). 18% stated that they do not use denosumab, whereas only 1% do not use zoledronic acid. The majority preferred to administer both zoledronic acid (70%) and denosumab (64%) every 4 weeks (**Figures 1b, c**). The most common time to initiate anti-resorptive therapy was on cycle 1, day 1 of anti-myeloma therapy (35%), although there was substantial heterogeneity of answers (**Figure 1d**).

**Figure 1:**
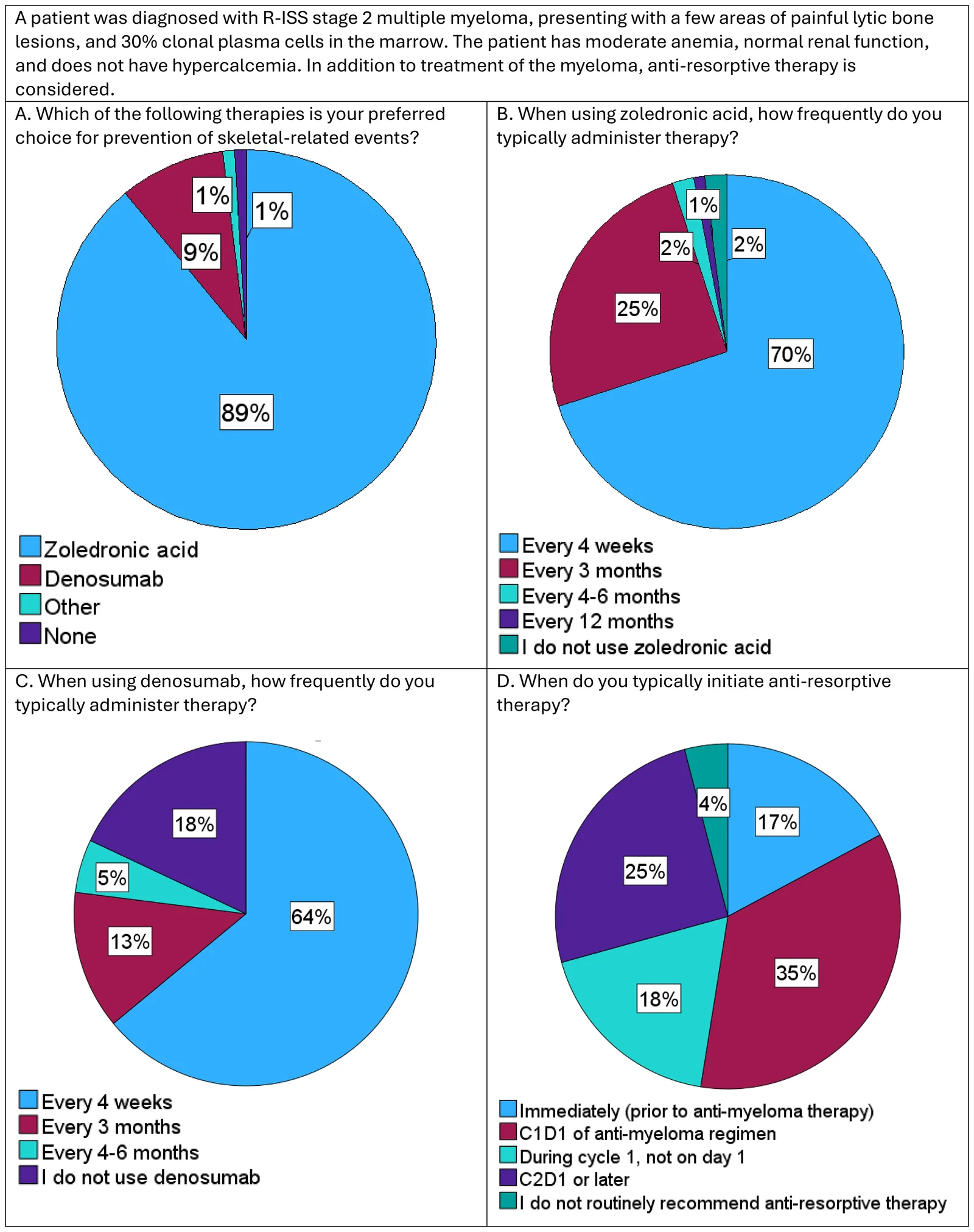
Choice of antiresorptive therapy in newly diagnosed myeloma. **(A)** Drug choice; **(B)** Frequency of zoledronic acid administration; **(C)** Frequency of denosumab administration; **(D)** Initiation time of antiresorptive therapy.

Respondents were prompted with a variety of scenarios different from the index patient, including absence of myeloma bone lesions, extensive bone lesions, pathological fractures, hypercalcemia, mild renal insufficiency, and severe renal insufficiency (eGFR <30) (**Figure 2**). In every scenario other than severe renal impairment, most respondents do not change their choice or frequency of anti-resorptive therapy. When considering patients with renal impairment, 73% of respondents (all but one of whom had chosen zoledronic acid as their preferred agent) stated that they would use a different drug for patients with severe renal insufficiency (eGFR <30), whereas 20% would choose a different drug for mild renal impairment. Among the 27 respondents who administer anti-resorptive therapy less every 3 months or greater, the presence of extensive lytic lesions, hypercalcemia, and pathological fracture(s) prompt more frequent administration of their chosen therapy in 63%, 59%, and 41%, respectively.

**Figure 2:**
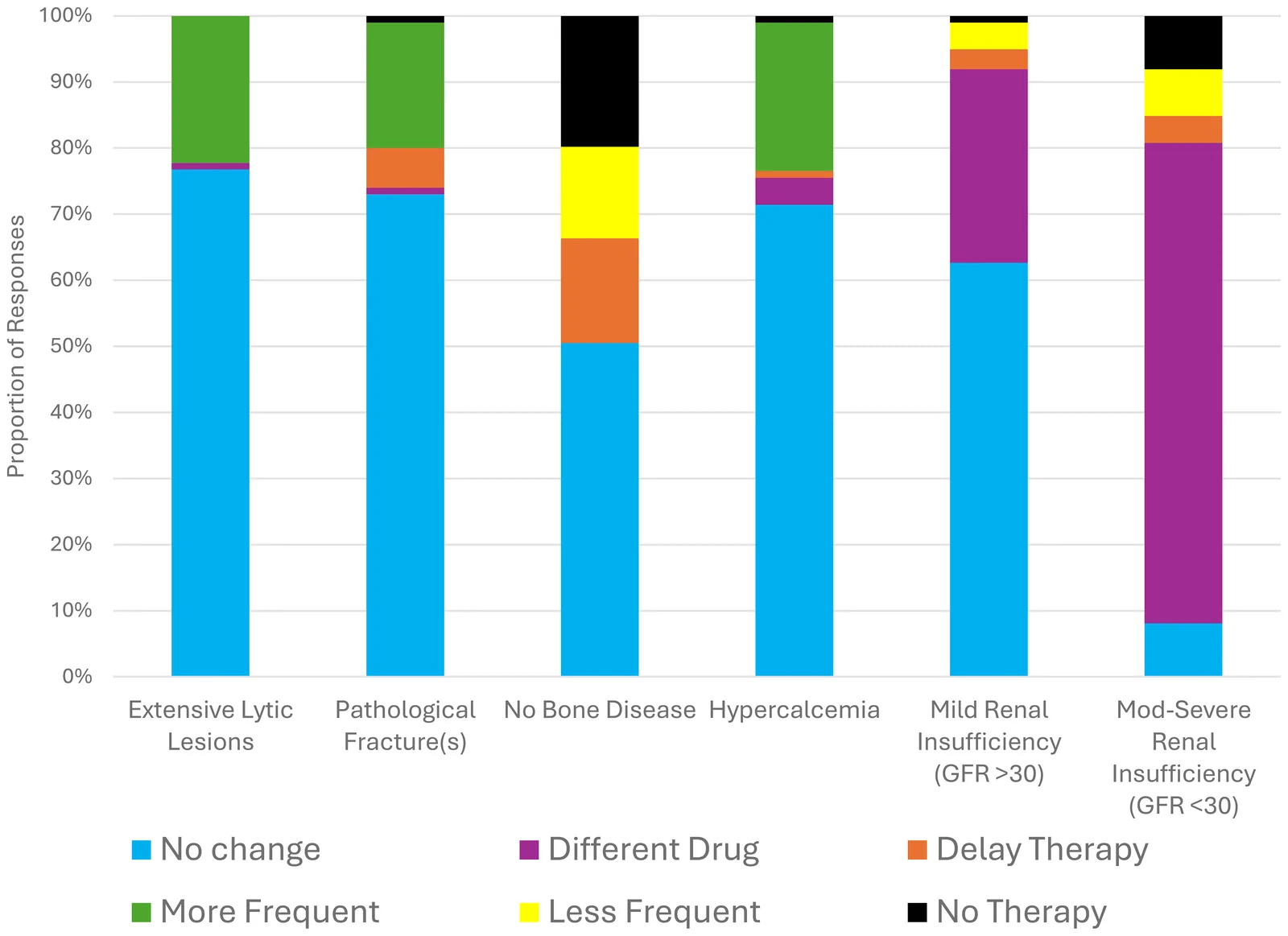
Modifications to choice of initial therapy based on clinical factors.

Practice patterns regarding dental considerations are shown in **Supplementary Figure S1**. More than half of respondents (54%) reported that they always request a dental evaluation prior to initiation of anti-resorptive therapy, whereas only 5% never do so. Factors that prompted a dental evaluation include poor dentition (30%) and time since the last dental evaluation (20%), whereas smoking history only impacted this decision for 8%. If a patient cannot afford a dental evaluation, most respondents (68%) would still administer on a case-by-case basis, with 15% administering to all patients, and 17% avoiding use in the absence of dental evaluation. For patients actively on anti-resorptive therapy who require a dental extraction, 40% recommended holding anti-resorptive therapy for 1 month prior to extraction, 49% hold for 3 months, and 8% hold for 6 months or greater. When osteonecrosis of the jaw (ONJ) is identified, 65% of respondents stated that they permanently discontinue antiresorptive therapy, with 35% deciding on a case-by-case basis.

Calcium supplements were always recommended by 53% of respondents, with 23% only doing so while patients were receiving anti-resorptive therapy, and 16% never recommending calcium supplements (**Supplementary Figure S2a**). Vitamin D was always recommended for use by 57% of respondents, with only 3% who never recommend its use. Regarding situational use, 13% recommend vitamin D concurrently with anti-resorptive therapy, 24% if they are vitamin D deficient, and 10% stating they had no preference about vitamin D supplementation in myeloma (**Supplementary Figure S2b**). For respondents who do recommend vitamin D, prescribing practices were varied. 38% prescribe cholecalciferol with specific instructions and doses, 18% administer ergocalciferol if vitamin D insufficiency or deficiency is identified, but a plurality (42%) of respondents did not have a preference about how to supplement vitamin D in patients with myeloma (**Supplementary Figure S2c**).

A majority of respondents (72%) administer anti-resorptive therapy for a total of 2 years among patients who are newly diagnosed and currently receiving maintenance and/or on observation. The next most common answers were indefinite and other duration (8% and 9%, respectively). However, duration and/or frequency of treatment was potentially impacted by several different clinical scenarios. Known osteoporosis or osteopenia (58%), extent of MBD at diagnosis (49%), and depth of response to anti-myeloma therapy (46%) were the most common reasons, excluding adverse effects, that impact recommended duration and/or frequency of anti-resorptive therapy (**Figure 3**). Despite osteoporosis or osteopenia being the most common reason to alter treatment decisions, only a minority of patients routinely order bone density (DEXA) scans, with 61% rarely ordering and 16% never ordering such imaging tests. When discontinuing denosumab, a plurality of respondents (49.5%) discontinue therapy abruptly, whereas 41.2% administer a dose of zoledronic acid to prevent rebound effects.

**Figure 3:**
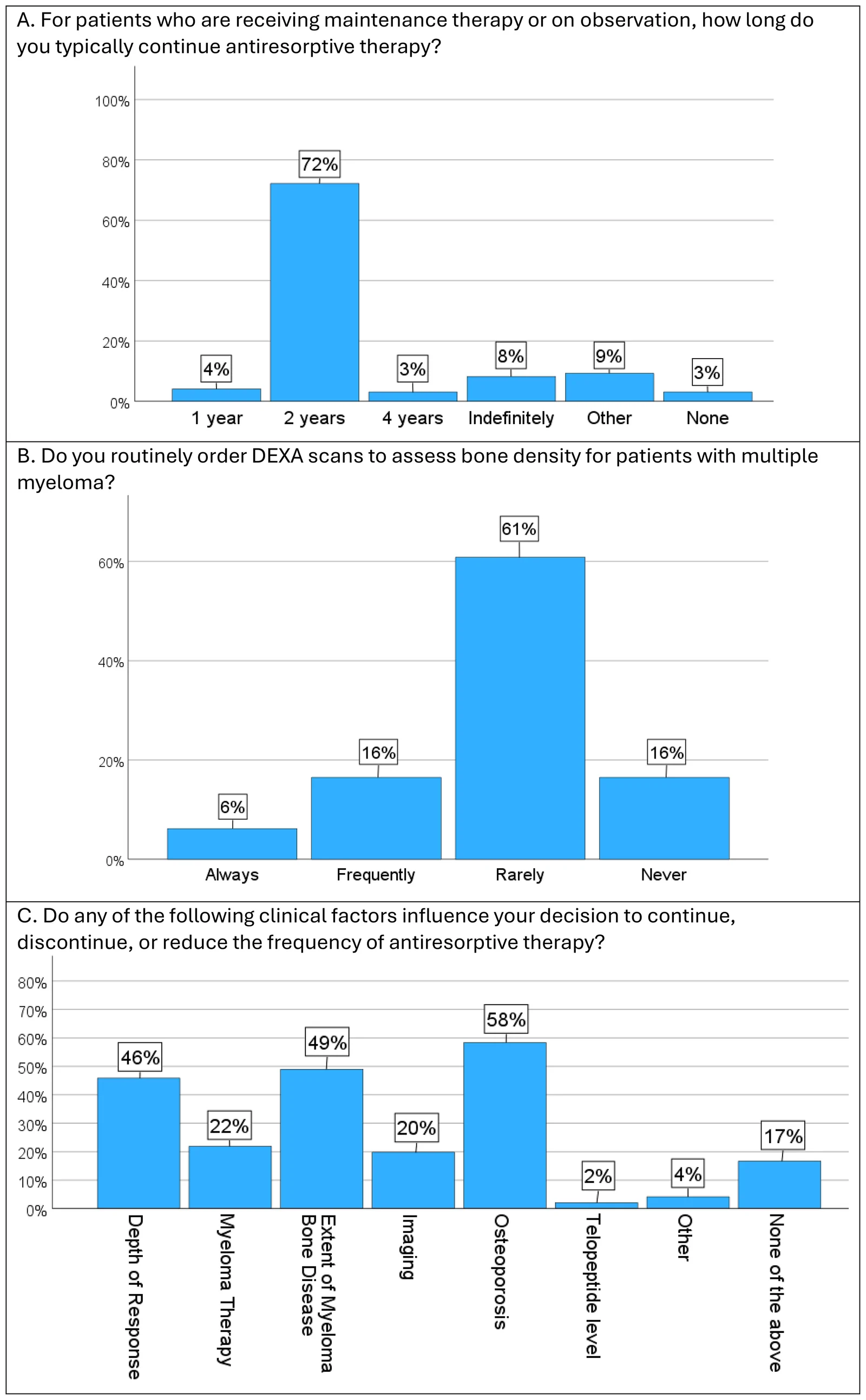
Duration and modifications of antiresorptive therapy. **(A)** Duration of antiresorptive therapy for patients who are receiving maintenance myeloma therap; **(B)** Use of bone density testing among patients receiving bone modifying agents for multiple myeloma; **(C)** Other clinical factors that influence duration of antiresporptive therapy for patients with newly diagnosed multiple myeloma.

Practice patterns for anti-resorptive use in the relapsed/refractory setting were highly variable, as shown in **Figure 4**. 45% of respondents stated that they continue or resume treatment, with 48% sometimes administering, and only 7% stating they do not administer treatment after disease progression. In this setting, 43% administer treatment every 3 months, 42% every 4 weeks, with a few responses for other or less frequent schedules. Regarding duration, the most common answer was 2 years (54%), whereas 28% administer for longer than 2 years, and 12% for only 1 year.

**Figure 4:**
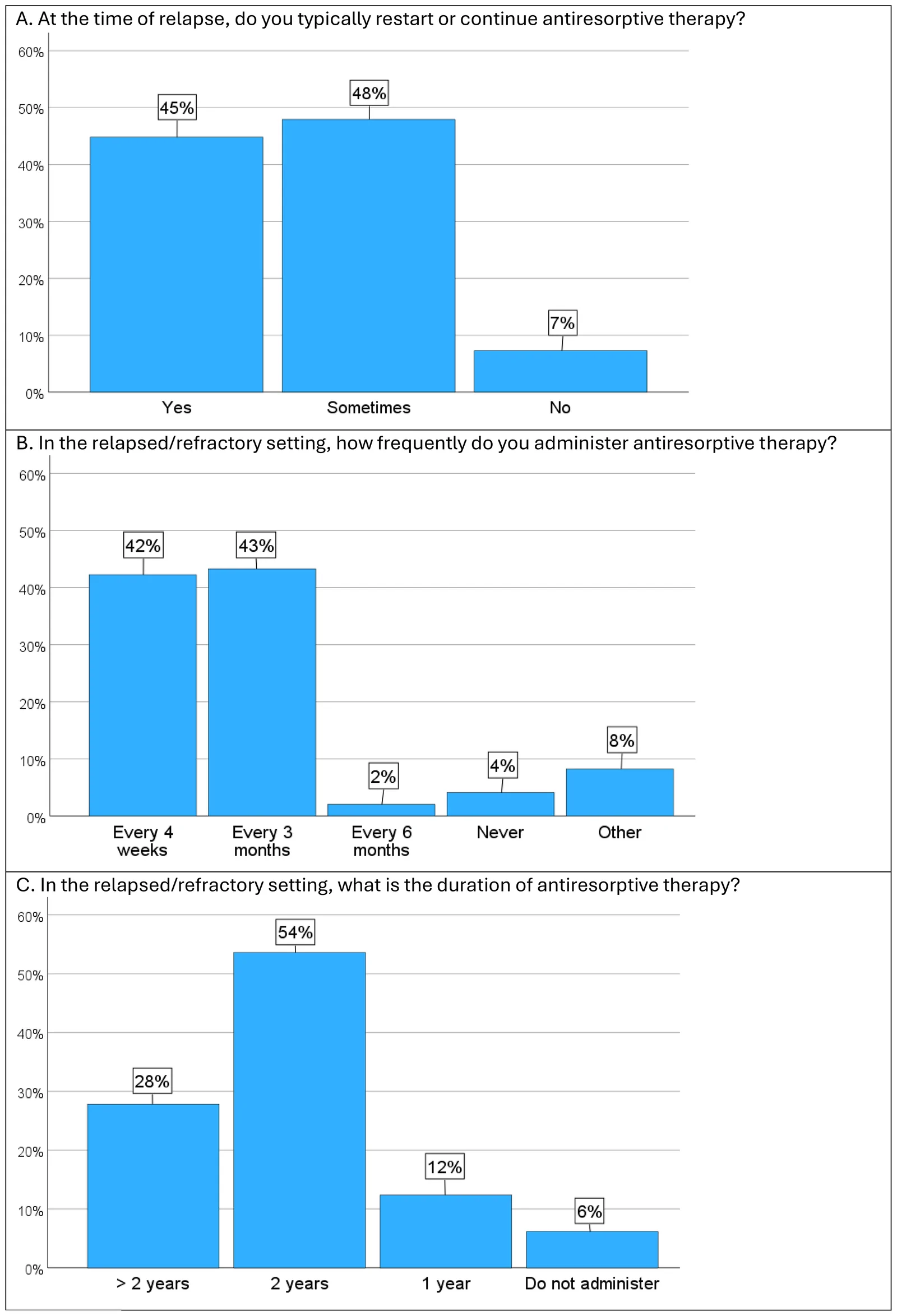
Management of bone disease in relapsed/refractory multiple myeloma. **(A)** Self-reported practice of whether antiresorptive therapy is given in the relapsed/refractory setting; **(B)** Frequency of administration of bone modifying agents in the relapsed/refractory setting; **(C)** Duration of antiresorptive therapy in relapsed/refractory myeloma.

## Discussion

In this multicenter survey of physicians, APPs, and pharmacists, from both academic and community settings, we report some of the most common practice patterns for managing MBD. While the results of this survey contain inherent selection bias based on the geographical and academic nature of affiliated sites, there are several important themes that emerged from our data.

Some practice patterns that had a relatively high degree of concordance among survey respondents. In the newly diagnosed setting, most respondents prefer Zoledronic acid, a dosing interval of every four weeks, and a duration of 2 years. Most respondents do not change their initial practice for anti-resorptive therapy in a variety of clinical scenarios, except for severe renal insufficiency, which prompted a majority to choose an alternative agent than Zoledronic acid.

Calcium and vitamin D supplements are recommended for all patients by most survey respondents, but specific recommendations were highly variable. More than half of respondents stated that they request a dental evaluation for all patients prior to initiation of anti-resorptive therapy, although only 17% would not be willing to use their selected therapy for patients unable to see a dentist due to cost or logistical reasons. For patients who require an invasive dental procedure, most respondents do not hold anti-resorptive therapy for longer than three months, although most respondents also do not feel comfortable resuming such therapy if the patient has a history of osteonecrosis of the jaw.

Practice patterns at relapse were highly variable. Despite near-unanimously reported use of anti-resorptive therapy in the newly diagnosed setting, a majority admit they do not routinely continue or resume therapy in the relapsed setting. Additionally, in contrast to the newly diagnosed setting, where 70% administer therapy every four weeks, a similar frequency of respondents administer every four weeks (42%) or every three months (43%) in the relapsed/refractory setting. While guidelines and expert opinions generally support use of anti-resorptive therapy in relapsed/refractory MM (16, 19), further research is required to help inform practice in this setting.

Taken together, these data show that while practice patterns are variable, the most common answers in this survey are concordant with the recommendations in the IMWG and ASCO guidelines (14, 16). A side-by-side comparison of these guideline recommendations and the findings from our survey are shown in **Table 2**. Some of the major areas where practice patterns are variable include more specific recommendations on how to supplement vitamin D, decision making processes when dental evaluation is not feasible, and when/how to tailor, discontinue, and resume anti-resorptive therapy. Several reasons could underly variability of practice and/or non-adherence to MBD guidelines, including a lack of knowledge, lack of feasibility to adhere to guidelines, and/or the perception that guidelines may not be relevant to individual patient care decisions. Our survey highlights that some of these areas such as resumption at relapse and tailoring of anti-resorptive therapy to depth of response or extent of bone disease may be areas that require more dedicated research and more specific recommendations in upcoming guidelines.

**Table 2:** Comparison of Bone-Modifying Agent Guidelines and Survey Practice.

Domain	IMWG (14)	ASCO (15)	NCCN Multiple Myeloma (REF)	EHA-EMN (16)	Our survey	Interpretation/knowledge gap
Initial antiresorptive treatment	ZA preferred in newly diagnosed MM, with or without MBD	BMA for active MM requiring systemic therapy: ZA/PAM, DEN alternative	ZA or PAM [category 1], or DEN for patients receiving primary MM therapy	Antiresorptive therapy for MM with osteolytic disease	89% ZA; 9% DEN	High consensus/guideline concordance. Initial agent selection is not the major knowledge gap.
No lytic bone disease	ZA recommended even without MBD; benefit less certain when modern imaging shows no disease	Supports BMA in active MM; also supports BP with osteopenia without lytic disease	BMA recommended for patients receiving primary MM therapy regardless of documented MBD	Treat if no lesions on conventional radiographs, but benefit unclear if imaging negative	51.5% no change; 20.2% no therapy; 16.2% delay; 14.1% reduce frequency	Important evidence/guideline gray zone. Variation may reflect uncertainty rather than nonadherence.
Initial dosing frequency	ZA every month	ZA every 3-4 weeks	Individualized every 1-3 months	ZA every month	70% ZA q4 weeks	Strong concordance at treatment initiation.
De-escalation after good response	After ≥12 months and ≥VGPR, consider reduced frequency or discontinuation	Every 3 months reasonable during maintenance	Individualized according to response and patient factors	≥VGPR: treatment 12-48 months, then individualized	46% report depth of response influencing duration/frequency	Potential practice gap, but guidelines differ on exactly when/how to de-escalate. Particularly relevant in modern deep responders.
Duration - deep response	≥12 months; after ≥VGPR consider de-escalation or discontinuation	Generally up to 2 years	Continue BMA for up to 2 years; continuation beyond 2 years based on clinical judgment	≥VGPR: 12-48 months	72% treat for 2 years	No single internationally agreed duration. The dominant survey practice aligns closely with ASCO and NCCN, while IMWG and EHA-EMN use more response-adapted approaches.
Duration - persistent disease or suboptimal response	Continued therapy generally supported	Continuation individualized according to ongoing skeletal morbidity	Continuation beyond 2 years based on clinical judgment	≤PR: monthly ZA for ≥4 years	Depth of response affects practice in only 46%	Important evolving evidence gap. EHA-EMN is more explicit about prolonged therapy for suboptimal response, whereas ASCO/NCCN are less prescriptive.
Severe renal impairment	DEN particularly attractive	ZA not recommended; DEN may be preferred because of lower renal toxicity	DEN preferred in renal disease; monitor renal function with BP	BP not recommended; DEN reasonable, with hypocalcemia caution	73% choose a different drug at eGFR <30	Directionally high concordance, but reporting only “different drug” prevents determining actual guideline concordance.
Mild renal impairment	BP dosing and monitoring individualized	Renal monitoring/dose modification	Dose adjustment and renal monitoring with bisphosphonates	Renal-function-dependent selection	20% change drug	Likely reasonable heterogeneity; actual drug selected would make this more interpretable.
Stopping denosumab	Discontinuation challenging because of rebound	Should not be stopped abruptly	Avoid abrupt discontinuation	Discontinuation requires mitigation because of rebound	49.5% stop abruptly; 41.2% give ZA	One of the clearest potential guideline-practice gaps in the survey.
Relapse	Reinitiate ZA if previously discontinued	Retreatment at relapse, particularly with new SREs	Reinitiate/continue based on clinical judgment, particularly with new or progressive MBD	Reinitiate ZA at relapse	45% routinely continue/resume; 48% sometimes; 7% do not	Major heterogeneity. “Sometimes” likely reflects clinical selection, but the survey did not capture rationale.
Calcium/Vitamin D	Recommended with antiresorptive treatment, with attention to renal function	Calcium/Vitamin D and laboratory monitoring recommended	Assess vitamin D status; calcium/vitamin D supplementation as appropriate	Supplementation/monitoring emphasized	Calcium always 53%; vitamin D always 57%; practices otherwise variable	Potential implementation gap; supplementation practices are less standardized than antiresorptive selection.
Dental/ONJ management	Preventive dental evaluation and avoidance/management of invasive procedures emphasized	Dental assessment/preventive dentistry recommended	Baseline dental examination strongly recommended; monitor for ONJ	Dental assessment; discontinue with ONJ and potentially restart after resolution	54% always obtain dental evaluation; extraction holds vary markedly; 65% permanently discontinue after ONJ	Mixture of implementation barriers and evidence uncertainty. Real-world dental access modifies practice.
Bone turnover markers	Historically proposed, but not established as routine strategy	Not central to routine treatment selection	Not a routine recommendation	Not a central routine-management recommendation	Very low use despite some clinicians de-escalating	Biomarker-directed dosing has not translated into routine clinical use.

ASCO, American Society of Clinical Oncology; BMA, bone modifying agent; BP, bisphosphonates (including zoledronic acid and pamidronate); DEN, denosumab; EHA-EMN, European Hematology Association-European Myeloma Network; IMWG, International Myeloma Working Group; MBD, myeloma bone disease; MM, multiple myeloma; mo, months; NCCN, National Comprehensive Cancer Network; ONJ, osteonecrosis of the jaw; PAM, pamidronate; PR, partial response; RI, renal impairment; SRE, skeletal-related event; VGPR, very good partial response; ZA, zoledronic acid.

Monitoring of biomarkers of bone turnover has been long proposed by the IMWG (20), but our survey indicates that this is rarely done in standard of care practice. Urine N-terminal telopeptide of collagen I (uNTX) levels are known to correlate well with the extent of MBD (21, 22). In the prospective Z-MARK study (23), uNTX monitoring was feasible and allowed for reduced frequency of zoledronic acid administration for patients with a reduction in bone turnover. In our survey, 26 respondents administer zoledronic acid at intervals longer than one month, but only 2 respondents replied that they check uNTX levels to help guide therapy. Presumably, many of these respondents assume that less frequent dosing is sufficient. This practice is indirectly supported by a large, randomized study, in which less frequent bisphosphonate use provided a similar benefit in reducing the risk of fractures related to bone tumors (24). However, myeloma constituted only 15% of patients in that study, and MBD is fundamentally different from osteoblastic metastatic disease from solid tumors. Dedicated studies are required to determine the optimal dose, schedule, and duration of anti-resorptive therapy and whether biomarkers should be used to tailor such therapy.

Our survey found low utilization of DEXA scans, which is not altogether surprising, given the paucity of data to support their routine use. Although one study showed that low lumbar bone density by DEXA scan was predictive of early vertebral fractures, DEXA was not useful as useful as telopeptide levels for monitoring of SRE risk over time (21). Furthermore, since most lytic lesions do not disappear, the validity of DEXA to evaluate changes in MBD is unknown, and the presence of lytic disease could lead to interpretation errors. Further research is needed to determine if any imaging modality can reliably help tailor MBD therapy.

The findings of this practice pattern analysis must be interpreted within its limitations. The survey sample, drawn from the BTCRC, represents a cohort primarily comprised of academic specialists and is not a random sample of all oncology practitioners. Consequently, the absolute percentages of specific practices may not be directly generalizable to the broader community setting. Because the survey captured management decisions but not the rationale underlying those decisions, it is difficult to determine whether observed practice variation reflects differences in institutional guidelines, interpretation of existing evidence, patient-specific considerations, or logistical factors. However, the variable practice patterns – particularly in relapsed myeloma, dental clearance barriers, and personalized approaches – are likely generalizable to different settings and constitute the study’s primary finding.

Other limitations include a lack of specificity or clarity in some of the survey questions. For instance, in the index case, the question asking about preferred frequency of administration did not specify whether that frequency was at the time of treatment initiation or if given on an ongoing basis. Likewise, when asked about modifications to the initial answer, use of a “different drug” was not specified, which reduces the power of conclusions that can be drawn from that answer. Our data also reflect the ideal practice patterns of the survey respondents, rather than what is done in actual clinical practice. Comparative analyses between different groups of respondents were unable to be performed due to inadequate sample size, and therefore this analysis remains descriptive in nature. Future endeavors should explore if specific groups of clinicians (e.g. physicians vs non-physicians) are more likely to deviate from guidelines or institutional norms and to evaluate whether clinicians truly adhere to their stated practice patterns in a busy clinical setting.

The fact that significant management differences exist among high-volume experts indicates that current guidelines provide helpful broad strokes but cannot address all clinical nuances due to limited evidence. Our data thus highlight a critical unmet need for more definitive studies and refined consensus recommendations to standardize care and optimize patient outcomes across all practice settings. Future efforts should prioritize prospective studies to define evidence-based best practices, ensuring that breakthroughs in myeloma treatment are matched by equally robust strategies to mitigate bone complications and sustain patient well-being.

## Data Availability

The original contributions presented in the study are included in the article/**Supplementary Material**, further inquiries can be directed to the corresponding author/s.
