# Supplementary material for: Practice patterns in myeloma bone disease: a multi-center survey
Source: Front Oncol. 2026 Oct 14;16:1948667. doi: 10.3389/fonc.2026.1948667 (PMC13713382; doi:10.3389/fonc.2026.1948667)
Supplement: Supplementary file 1 [file DataSheet1.pdf]

Supplementary Appendix

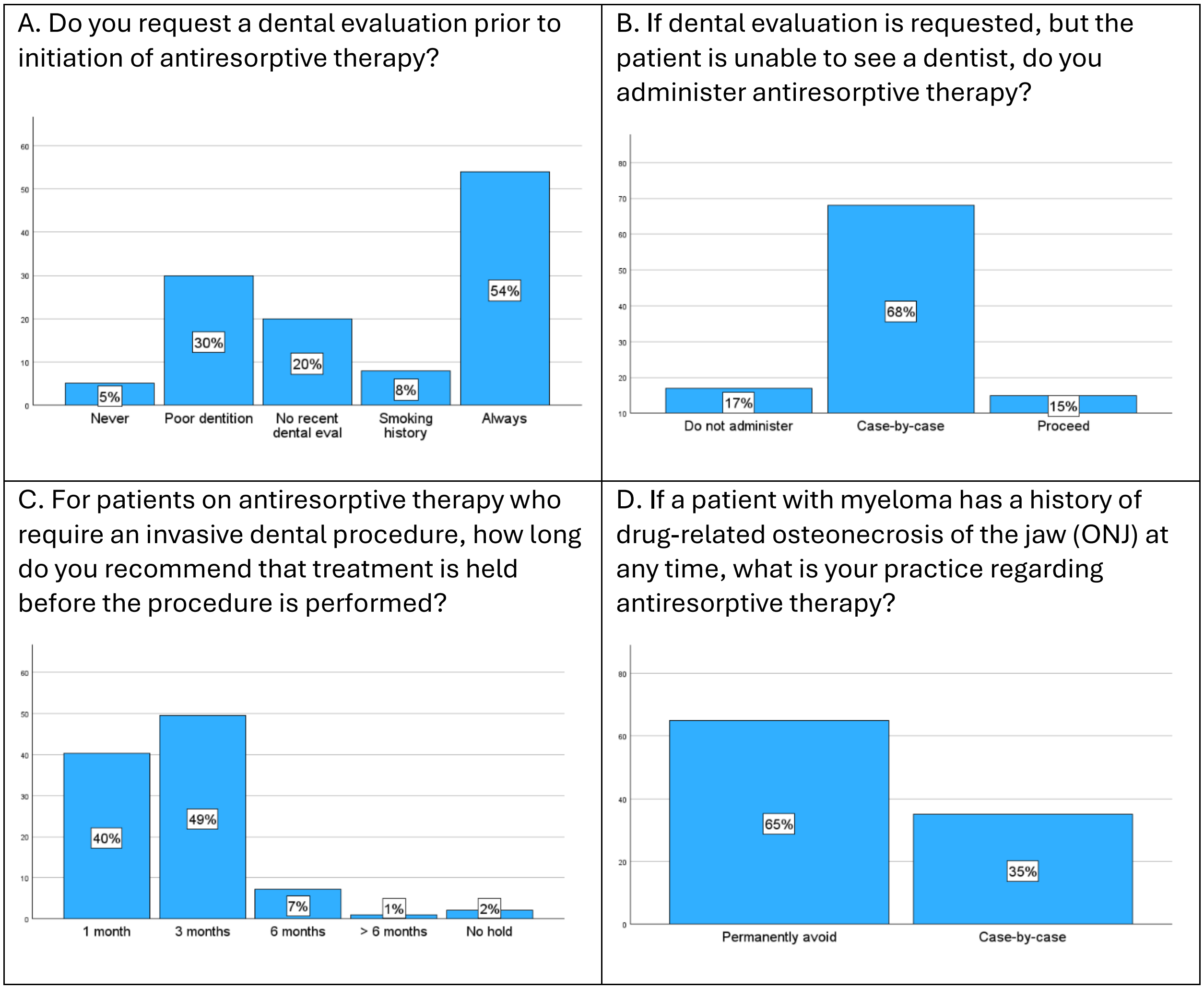

Figure S1. Dental considerations with anti-resorptive therapy.

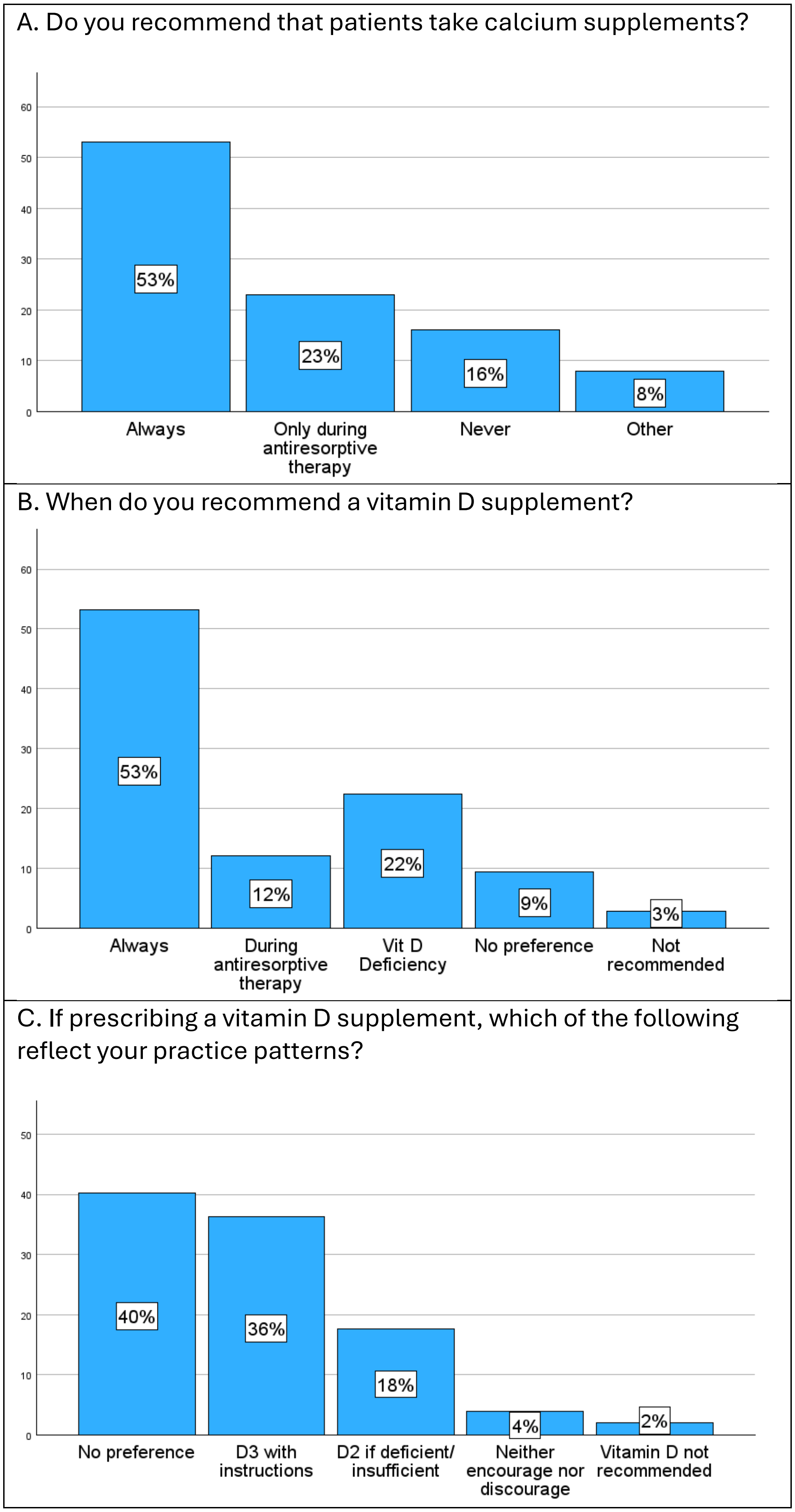

Figure S2. Calcium and Vitamin D Supplementation

Default Question Block

Hello,

Thank you for your interest in this survey. We are interested in learning about your practice patterns regarding supportive care for myeloma bone disease. The purpose of this survey is to accumulate information about practice patterns regarding supportive care for myeloma bone disease, and the results will be used for research purposes only.

The survey is intended for clinicians who:
- Have an active role in making clinical treatment decisions for patients with multiple myeloma
- Work at a medical center or clinic that is affiliated with a university that is a part of the Big Ten Cancer Research Consortium. This may include a central hospital location, satellite campuses, or other affiliated locations within the health system.

Survey participants will not directly benefit from this study, but the primary study member at each participating university system will be offered authorship on any publications that may result from the survey. Additionally, we hope that the results will help to inform the field about how to generalize myeloma bone health research to standard clinical practice. All responses will be anonymized, and there are no expected risks associated with completing this survey.

If you meet the above criteria and would like to participate, please proceed to answer the following questions. The survey consists of 24 brief questions and is anticipated to take no more than 5 minutes to complete.

By completing this survey, you are indicating that you meet the eligibility criteria listed above and that you have provided informed consent to participate in the research study.

Your participation in this survey is completely voluntary, and you may withdraw consent at any time by contacting Timothy Schmidt, MD, at tschmidt@medicine.wisc.edu

- ○ I agree to participate in this survey
- ○ I decline participation in this survey

What is your professional role?

- ○ Physician
- ○ Advanced Practice Provider, Nurse Practitioner or Physician Assistant
- ○ Pharmacist
- ○ Other (please specify) ____________

What is your primary practice setting?

- ○ Academic or University hospital
- ○ Academic-affiliated community practice
- ○ Community/private practice
- ○ Other ____________

How long have you been practicing independently?

- ○ 0-5 years
- ○ 5-10 years
- ○ 10-20 years
- ○ 20+ years

How many patients with multiple myeloma do you see annually?

- ○ 0-5
- ○ 5-20

- 20-50
- 50-100
- 100+

With which Big Ten Cancer Research Consortium institution are you affiliated?

- Michigan State University
- Ohio State University
- Penn State University
- Purdue University
- Rutgers University
- University of Illinois
- University of Indiana
- University of Iowa
- University of Maryland
- University of Michigan
- University of Minnesota
- University of Nebraska
- University of Wisconsin

Block 1

A patient was diagnosed with R-ISS stage 2 multiple myeloma, presenting with a few areas of painful lytic bone lesions, and 30% clonal plasma cells in the marrow. The patient has moderate anemia, normal renal function, and does not have hypercalcemia. In addition to treatment of the myeloma, anti-resorptive therapy is considered.

Which of the following therapies is your preferred choice for prevention of skeletal-related events?

- Zoledronic acid
- Denosumab
- Other ______
- None

When using zoledronic acid, how frequently do you typically administer therapy?

- Every 4 weeks
- Every 3 months
- Every 4-6 months
- Every 12 months
- I do not use zoledronic acid

When using denosumab, how frequently do you typically administer therapy?

- Every 4 weeks
- Every 3 months
- Every 4-6 months
- Every 12 months
- I do not use denosumab

When do you typically initiate anti-resorptive therapy?

- Immediately (prior to anti-myeloma therapy)
- C1D1 of anti-myeloma regimen
- During cycle 1, not on day 1
- C2D1 or later
- I do not routinely recommend anti-resorptive therapy

Compared to the initial case, would you change anything about your recommended anti-resorptive therapy in any of the following circumstances?

	No change	Use a different drug	Delay initiation of therapy	Increase administration frequency	Decrease administration frequency	Would not use any therapy
Extensive lytic lesions	☐	☐	☐	☐	☐	☐
Pathological fracture, including compression fracture(s)	☐	☐	☐	☐	☐	☐
Absence of myeloma bone disease on cross-sectional imaging	☐	☐	☐	☐	☐	☐
Hypercalcemia at diagnosis	☐	☐	☐	☐	☐	☐
Mild-moderate renal insufficiency (GFR >30)	☐	☐	☐	☐	☐	☐
Severe renal insufficiency (GFR <30)	☐	☐	☐	☐	☐	☐

Do you request a dental evaluation prior to initiation of anti-resorptive therapy?

- ☐ Never
- ☐ If there is poor dentition on inspection of the oral cavity
- ☐ If it has been longer than a certain time period since the last dental evaluation
- ☐ If patient is a current or recent smoker
- ☐ For all patients, regardless of the date of their last dental evaluation

If dental evaluation is requested, but the patient is unable to see a dentist (due to cost,

logistics, etc), what is your typical practice?

- ○ I do not administer a bisphosphonate or RANKL inhibitor
- ○ I will consider anti-resorptive therapy on a case-by-case basis
- ○ I proceed with anti-resorptive therapy

Do you recommend that patients take calcium supplements?

- ○ Always (allowing exceptions for contraindications or AEs)
- ○ Only while concurrently receiving antiresorptive therapy
- ○ Never
- ○ Other ______

When do you recommend a vitamin D supplement?

- ☐ Always (allowing exceptions for contraindications or AEs)
- ☐ In patients who are concurrently receiving antiresorptive therapy
- ☐ If patient is vitamin D deficient or insufficient
- ☐ I do not have a preference regarding vitamin D supplementation and myeloma
- ☐ I do not recommend vitamin D supplementation

If prescribing a vitamin D supplement, which of the following reflect your practice patterns?

- ☐ I do not have a preference regarding how vitamin D is supplemented in myeloma
- ☐ Patients are given specific instructions regarding dose and frequency of cholecalciferol (D3) supplementation
- ☐ I check vitamin D levels and prescribe ergocalciferol (D2) for repletion when insufficiency or deficiency is identified
- ☐ Patients are neither encouraged or discouraged from taking vitamin D
- ☐ I do not recommend vitamin D supplementation

For patients on anti-resorptive therapy who require an invasive dental procedure (for instance, pulling a tooth), how long do you recommend that treatment is held before the procedure is performed?

- 1 month
- 3 months
- 6 months
- Longer than 6 months
- I do not hold therapy for any duration of time before dental procedures

For patients who are receiving maintenance therapy or on observation, how long do you typically continue anti-resorptive therapy?

- 1 year
- 2 years
- 4 years
- Indefinitely
- Other
- I do not use anti-resorptive therapy on an ongoing basis

Do any of the following clinical factors influence your decision to continue, discontinue, or reduce the frequency of antiresorptive therapy?

- [ ] Response to anti-myeloma therapy (i.e. achievment of VGPR or CR)
- [ ] Concurrent use of anti-myeloma therapy (i.e. steroids)
- [ ] Extent of myeloma bone disease at diagnosis
- [ ] Residual uptake on functional imaging (i.e. PET scan)
- [ ] Known osteoporosis and/or osteopenia
- [ ] Urine telopeptide level
- [ ] Other

- [ ] None of the above

Do you routinely order DEXA scans to assess bone density for patients with multiple myeloma?

- ○ Always
- ○ Frequently
- ○ Rarely
- ○ Never

If a patient is on denosumab and a decision is made to discontinue therapy, what is your recommendation regarding how to discontinue therapy?

- ○ Discontinue therapy and monitor symptoms and laboratory parameters
- ○ Administer a dose of zoledronic acid at a specified interval (i.e. 6 months) after the last dose of denosumab
- ○ I do not discontinue denosumab once a patient has been started on it
- ○ Other ________

At the time of relapse, do you typically restart or continue anti-resorptive therapy?

- ○ Yes
- ○ Sometimes
- ○ No

In the relapsed and/or refractory setting, how frequently do you administer antiresorptive therapy?

- ○ Every 4 weeks
- ○ Every 3 months
- ○ Every 6 months

- Never
- Other ______

In the relapsed/refractory setting, assuming there are no contraindications, what is the duration of antiresorptive therapy?

- Longer than 2 years
- 2 years
- 1 year
- I do not administer antiresorptive therapy in the relapsed/refractory setting

If a patient with myeloma has a history of drug-related osteonecrosis of the jaw (ONJ) at any time, what is your practice regarding antiresorptive therapy?

- I permanently avoid antiresorptive therapy if a patient has a history of ONJ
- Antiresorptive therapy may be administered and/or resumed on a case-by-case basis
- Antiresorptive therapy is administered regardless of a history of ONJ

Powered by Qualtrics
